# Genetic Variants Correlate With Better Processing Speed

**DOI:** 10.1093/geroni/igab046.623

**Published:** 2021-12-17

**Authors:** Anastasia Gurinovich, Kaare Christensen, Marianne Nygaard, Jonas Mengel-From, Stacy Andersen, Thomas Perls, Paola Sebastiani, Zeyuan Song

**Affiliations:** 1 Boston University, Boston, Massachusetts, United States; 2 Department of Public Health, University of Southern Denmark, Odense, Syddanmark, Denmark; 3 Boston University School of Medicine, Boston, Massachusetts, United States; 4 Tufts Medical Center, Physician Organization, BOSTON, Massachusetts, United States; 5 Boston University School of Public Health, Boston, Massachusetts, United States

## Abstract

Some cognitive abilities, such as vocabulary, are resilient to brain aging, while others such as conceptual reasoning, memory, and processing speed, decline with age and their rate of decline is genetically regulated. Despite the strong genetic heritability of processing speed assessed by the digit symbol substitution test (DSST), previous studies have failed to identify robust common genetic variants associated with this test. The Long Life Family Study (LLFS) includes long lived individuals and their family members who maintain good DSST scores as they age and who may carry variants associated with better DSST. We therefore conducted a genome-wide association study (GWAS) of DSST in LLFS using ~15M genetic variants imputed to the HRC panel of 64,940 haplotypes with 39,635,008 sites and replicated the findings using genetic data imputed to the 1000 Genomes phase 3 reference panel combining two Danish cohorts: the Middle Aged Danish Twins and the Longitudinal Study of Aging Danish Twins. The GWAS in LLFS discovered 20 rare genetic variants reaching genome-wide significance (p-value < 5x10-8), including 18 variants associated with better processing speed with large effect size. The genetic associations of rs7623455, rs9821776, rs9821587, rs78704059 in chromosome 3 were replicated in the combined Danish cohort. These genetic variants tagged two hormone receptor related genes, THRB and RARB, both related to cognitive aging. Further gene-based tests in LLFS confirmed that these two genes have protective variants associated with better processing speed.

